# Insights to Genetic Characterization Tools for Epidemiological Tracking of *Francisella tularensis* in Sweden

**DOI:** 10.1371/journal.pone.0112167

**Published:** 2014-11-17

**Authors:** Tara Wahab, Dawn N. Birdsell, Marika Hjertqvist, Cedar L. Mitchell, David M. Wagner, Paul S. Keim, Ingela Hedenström, Sven Löfdahl

**Affiliations:** 1 Public Health Agency of Sweden, Department of Microbiology, Stockholm, Sweden; 2 Northern Arizona University, Center for Microbial Genetics and Genomics, Flagstaff, AZ, United States of America; University of Louisville, United States of America

## Abstract

Tularaemia, caused by the bacterium *Francisella tularensis*, is endemic in Sweden and is poorly understood. The aim of this study was to evaluate the effectiveness of three different genetic typing systems to link a genetic type to the source and place of tularemia infection in Sweden. Canonical single nucleotide polymorphisms (canSNPs), MLVA including five variable number of tandem repeat loci and *PmeI*-PFGE were tested on 127 *F. tularensis* positive specimens collected from Swedish case-patients. All three typing methods identified two major genetic groups with near-perfect agreement. Higher genetic resolution was obtained with canSNP and MLVA compared to PFGE; *F. tularensis* samples were first assigned into ten phylogroups based on canSNPs followed by 33 unique MLVA types. Phylogroups were geographically analysed to reveal complex phylogeographic patterns in Sweden. The extensive phylogenetic diversity found within individual counties posed a challenge to linking specific genetic types with specific geographic locations. Despite this, a single phylogroup (B.22), defined by a SNP marker specific to a lone Swedish sequenced strain, did link genetic type with a likely geographic place. This result suggests that SNP markers, highly specific to a particular reference genome, may be found most frequently among samples recovered from the same location where the reference genome originated. This insight compels us to consider whole-genome sequencing (WGS) as the appropriate tool for effectively linking specific genetic type to geography. Comparing the WGS of an unknown sample to WGS databases of archived Swedish strains maximizes the likelihood of revealing those rare geographically informative SNPs.

## Introduction

*Francisella tularensis* is a highly virulent, facultative intracellular pathogen, which causes tularaemia in humans and animals [1]. Transmission occurs via arthropod bites (mosquitoes and ticks), inhalation of contaminated dust, or ingestion of contaminated food or water. Tularaemia is endemic in Sweden and has been reported since 1931 causing between 100–700 infections annually [2]. The most common clinical form is ulceroglandular tularaemia and all reported human cases of tularaemia in Sweden have been caused by *F. tularensis* subspecies *holarctica* (type B). The disease occurs with a seasonal pattern – being especially prevalent in summer and autumn. It is most common in Northern and Middle Sweden but during the last decade the infection has spread further south (SmiNet2, Swedish National Surveillance System).

Recently, several molecular typing methods have been utilized in studies of genetic population structure of *F. tularensis* including: PFGE, whole genome microarrays, multi-locus variable-number of tandem repeat (VNTR) analysis (MLVA), insertion-deletion markers (INDELs), and single nucleotide polymorphism (SNP) [3]–[12]. SNPs are point mutations that are evolutionarily stable in clonally reproducing organisms such as *F. tularensis* [12]. This stability makes them useful for classifying bacterial populations into specific genetic groups where phylogenetic relationships among groups can be accurately inferred [13]. Several studies have constructed a global phylogenetic tree using canSNPs, subdividing the subspecies *holarctica* samples into 30 subpopulations found worldwide [6], [8], [11], [12], [14]. MLVA has been very useful for several bacterial species, which, like *F. tularensis*, have little genomic variation [15], [16]. MLVA analysis using 25 VNTR markers on 139 *F. tularensis* subspecies *holarctica* samples from North America, Europe and Asia, revealed five subpopulations, which with one exception were found in several well separated geographic areas [7].

The aim of present study was to evaluate canSNPs, MLVA, and *PmeI-*PFGE for practical uses in epidemiological investigation of *F. tularensis* infections in Sweden and in combination with geomapping to possibly predict sources of infection and reservoirs.

## Results

### Molecular typing

**Figure 1:**
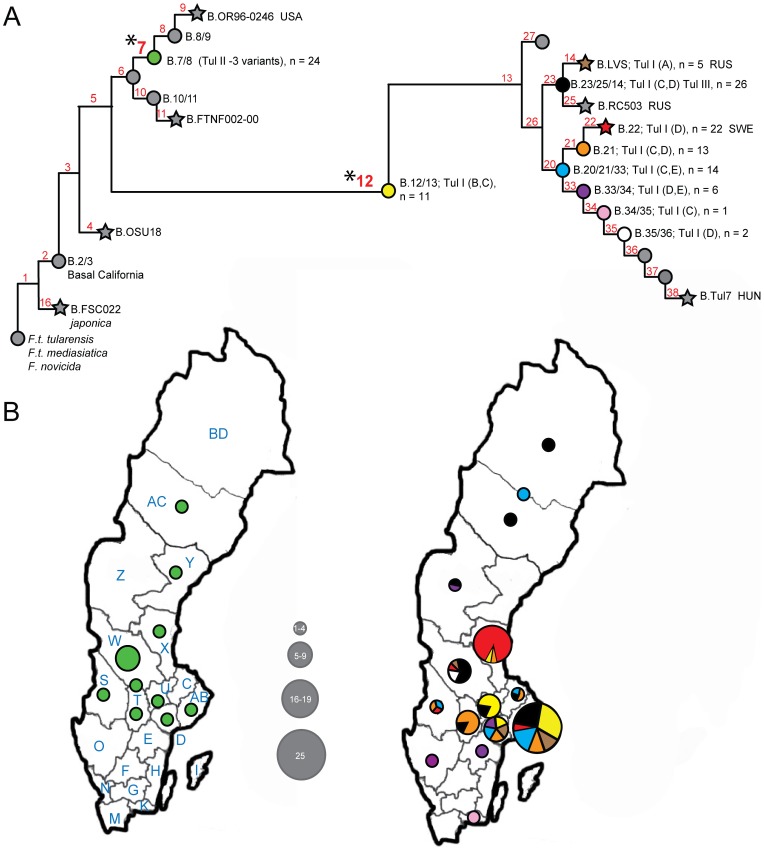
Detailed phylogeographic patterns of 103 human patient samples from Sweden. A) Existing global canSNP phylogeny of *F. tularensis* subsp. *holarctica* (Gyuranecz et al) wherein subgroups are indicated as circles and reference strains as stars. Gray coloration indicates subgroups not identified in this study. Noted for each mapped subgroup are the MLVA genotypes and n values (i.e. number of strains). The country code for each sequence genome is indicated: HUN, RUS, SWE, USA. B) Geographic distribution of groups on separate maps of Sweden based on membership to one of the two major subgroups (*B.7/8 and *B.12). County code is indicated on the map (left). Total number of samples per county is represented by the circle size. Multiple subgroups within a single county are represented proportionally on a pie chart comprised of the colors corresponding to subgroups.

To evaluate the effectiveness of SNP, MLVA and PFGE typing as methods for tracing source and place of infection within an endemic country, we analysed 127 clinical specimens (Table S1). We discovered that all three typing methods identified two major genetic groups with near-perfect agreement. Six samples were removed from canSNP analysis due to DNA quality issues. Analysis with canSNPs revealed that all *F. tularensis* samples were assigned to two major groups: B.7/8 (n = 24) and B.12 (n = 97) (Figure 1A). The proportion of B.12 phylogroup to B.7/8 was in agreement with previous publication [8]. MLVA analysis resulted in two major clusters, Tul-I and Tul-II that corresponded with the B.12 and B.7/8 lineages, respectively (Table S1; Figure 1A). Two samples falling within the B.12 lineage formed a distinct MLVA cluster, Tul-III (Table S1). The two major MLVA clusters (Tul-I and Tul-II) were based on differences in the two less discriminating markers, Ft-M22 and Ft-M24, with only two allelic variants each. Tul-III MLVA cluster differed from all other MLVA types in the allele combination of VNTR markers Ft-M24 and Ft-M22 with 1 and 4 copies, respectively. Two other canSNP groups (B.4 and B.10) that were not found in this study have been previously reported in Sweden [8], [11], [12]. However, samples of these phylogroups are rather rare in Sweden so their lack of presence in this study may be due to our smaller sample size.

**Figure 2:**
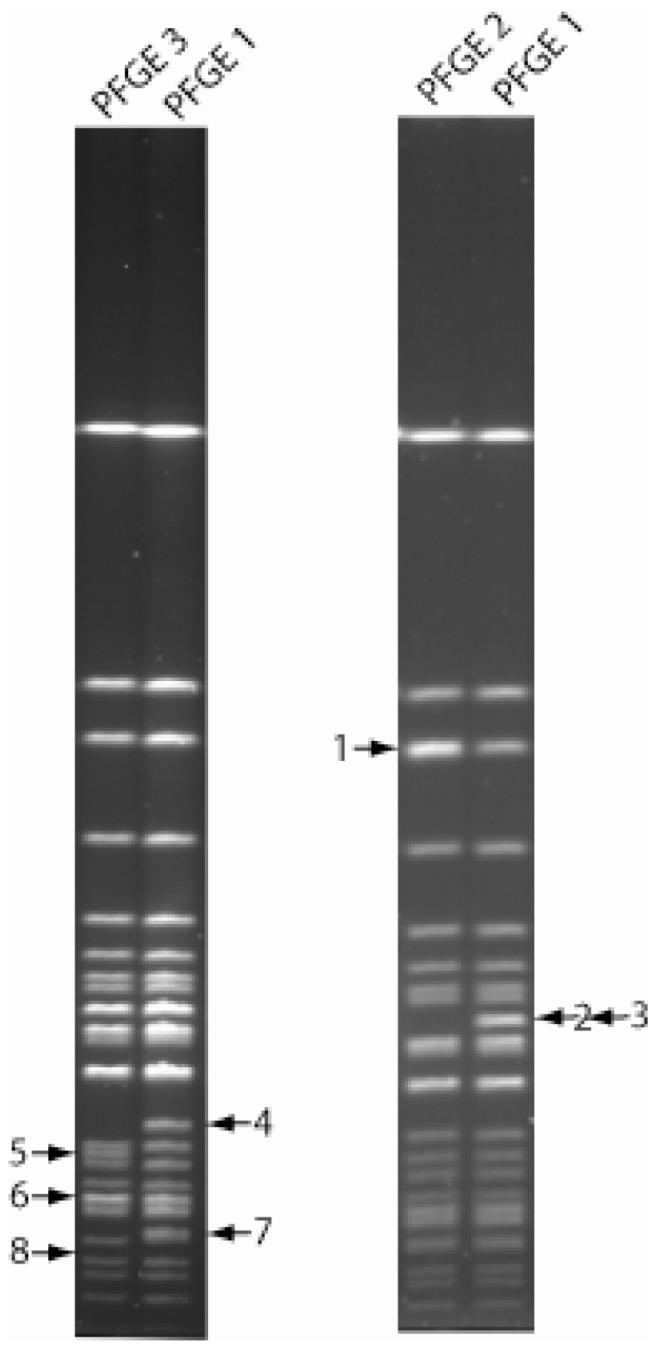
*PmeI* pulsed-field gel electrophoresis (PFGE) patterns for *F. tularensis* subsp. *holarectica*. Polymorphic band position 1 consists of two fragments in PFGE type 2. Polymorphic band position 2 and 3 consist of two fragments at the same position, both missing in PFGE type 2. Polymorphic band position 6 consists of two fragments in PFGE type 3.

PFGE analysis of the 124 clinical samples revealed three different but rather similar PFGE profiles, types 1–3 (Figure 2). Comparison of PFGE data to MLVA and canSNP data (Table S1) revealed that PFGE types 1 and 3 corresponded to the two major canSNP groups B.12 (Tul-I) and B.7/8 (Tul-II), respectively (Table S1). MLVA cluster Tul-III corresponded with PFGE type 2. Taken together, canSNP, MLVA, and PFGE were in general agreement when classifying samples into major groups.

Higher genetic resolution was obtained with SNP and MLVA analyses compared to PFGE. Samples within the B.12 SNP lineage were further classified into one of nine minor phylogroups (B.12/13, n = 11; B.14, n = 5; B.20/21/33, n = 13; B.21/22, n = 13; B.22, n = 22; B.23/14/25, n = 24, B.33/34, n = 6; B.34/35, n = 1; and B.35/36, n = 2) (Figure 1A; Table S1) using SNP-signatures previously described [6], [11], [12]. Combining all five VNTR markers resulted in 33 unique MLVA subtypes wherein 17 unique subtypes were the result of variation at the Ft-M3 locus, which was the most rapidly mutating marker. Tul-I samples were assigned into one of five smaller subgroups (A–E), with each alpha code genotype further divided into Ft-M3 subtypes. Tul-II samples were less diverse than Tul-I, resulting in 3 unique MLVA-types referred to as #8, #10, and #33. The number of alleles of the five markers varied exhibiting moderate to high diversity (D) ranging from 0.15–0.90 as previous described [7] (Table 1).

**Table 1:** VNTR markers.

VNTR marker	Repeat size (nt)	No. of repeats*		No. of alleles*	Diversity* †	Inside orf††
		Min	Max			
Ft-M3	9	8	25	17	0.90	Yes
Ft-M6	21	4	6	3	0.61	Yes
Ft-M20	12	2	4	3	0.15	Yes
Ft-M22	6	3	4	2	0.34	Yes
Ft-M24	21	1	2	2	0.36	No

*Data obtained in this study.

† The individual marker diversity (D) was calculated as D = [1-∑(allele frequency)^2^].

†† Location within an open reading frame.

At this higher resolution level, MLVA and canSNP assignment of samples were not in consistent agreement. Samples within identical MLVA genotype did not fall into a single canSNP group, but rather fell into multiple SNP phylogroups (Figure 1A; Table S1). Several canSNP groups contained multiple MLVA genotypes (Figure 1A; Table S1). This lack of grouping agreement is due to the type of genetic markers (SNPs or VNTRs) targeted by each typing system. SNP mutations are highly stable and therefore are informative of the genetic relationships among groups of isolates [13]. MLVA typing is based on VNTR loci that can be prone to mutational instability and, therefore, often result in homoplasy, making inferences about relationships among groups of isolates unreliable. When SNP analysis and MLVA are used in a hierarchical scheme, accurate genetic relatedness among SNP-defined groups of *F. tularensis* samples can be confidently known (Figure 1A) [12], [13] and the MLVA data provided finer levels of discrimination among samples within the same phylogroup.

### Geographic distribution of SNP groups

A highly complex geographic pattern among the phylogroups exists at a national and regional scale in Sweden (Figure 1). Our data present a pattern of wide distribution of multiple phylogroups that are found in multiple relatively distant counties in Sweden. Samples from both major groups (B.7/8 and B.12) are represented in middle and northern Sweden, with the highest density found in middle Sweden. Southern Sweden is sparse and is represented by samples classified in closely related terminal phylogroups within B.12 lineage (B.33/34 and B.34/35). Nearly all phylogroups identified in this study are present in middle Sweden, making this region the center of diversity, as previously described [8].

As a consequence of wide distribution of multiple phylogroups, extensive phylogenetic diversity was found within individual counties in middle Sweden. The richest diversity is found in Stockholm area (Figure 1B, county AB), representing seven diverse phylogroups, each with multiple MLVA subtypes. Örebro, a city in middle Sweden, was co-localized with 3 distinct phylogroups and MLVA subtypes (Table S1 and Figure 1, county T). Most samples appeared to cluster along major waterways, such as rivers and coastal areas of the Swedish east coast, the Baltic Sea. There was no obvious geographical correlation to age and gender of the patients (data not shown), but adults were significantly more commonly infected than children. Water bodies, like the Dalälven river, appears to be linked to multiple phylogroups that are diverse (Figure 1B, county W).

In contrast, Ljusnan river (county X in Figure 1B) is linked to seventeen case-patient samples that are highly genetically similar despite the 12 year span (1995–2006). All samples, except one, belonged to subgroup B.22 MLVA D4. The remaining sample fell in the B.21/22 subgroup, which is the closest relative to B.22 samples. Despite this pattern, 3 B.22 samples were found in 3 other nearby counties in middle Sweden, including Stockholm (Figure 1B), which may reflect recent dispersal from the founding source. Taken together, small regional sections or water bodies are co-localized with multiple phylogroups with great genetic diversity.

## Discussion

The complex phylogeographic pattern of tularemia distribution in Sweden poses a great challenge to accurately identify a source and place of infection for any given *F. tularensis* specimen within Sweden (Figure 1). Our attempts to correlate geographic origin of the samples to specific genetic types did not generate a clear cut result (Figure 1B) despite employing a canSNP typing scheme that provided higher genetic resolution [6] than in the study performed by Karlsson et al 2013 and colleagues. This SNP typing scheme allowed us to place 121 clinical specimens, collected throughout Sweden over a span of 16 years, on the existing global phylogenetic tree for *F. tularensis* subsp. *holarctica* (Figure 1A) [6]. All phylogroups identified in Sweden have also been found in distant nations abroad, except B.22, which is restricted to Sweden. Given this broader dispersal range, it is no surprise that samples within these specific phylogroups are broadly dispersed in Sweden across multiple counties and not restricted to a small geographic region. The broad distribution of closely related phylogroups is central to the argument that *F. tularensis* is a rapidly dispersed organism. The basis for this rapid dispersal remains unclear, but recent reports suggest dispersal could be facilitated by the migration of birds [19], [20].

Despite the phylogeographic complexity, patterns emerge that suggest the possibility of identifying SNP markers that could be meaningfully linked to geographic regions or at least narrow the geographic range of possible places. An example of this is the SNP for B.22 phylogroup, which is comprised of samples found only in Sweden [6], [11], [12]. It is interesting to note that unlike all other phylogroups in this study, B.22 SNP was identified from a Swedish genome (FSC200) linked to the Ljusnan river in county X (Figure 1). This B.22 SNP was highly specific to FSC200 genome. All other phylogroups are defined by SNPs discovered from genomes found in other nations (USA, Russia, and Hungary) [6], [11], [12]. Most case-patients (16/17) linked to the Ljusnan river, collected over a 12 year time frame (1995–2006) (Figure 1B), typed as B.22 MLVA D4 (Table S1). The striking genetic similarity suggests that they all recently emerged from a common ancestor. The temporal pattern rules out a single outbreak season and provides insight into the stability of this genotype. Taken together, these data suggest that the B.22 D4 genotype originated from a common ecological niche that spanned the approximately 70 kilometres distance of the river. Intense localized sampling of beaver populations may verify this hypothesis. Beavers, which are common on this river, could be a viable source given that these semi-aquatic rodent species have shown evidence of seroconversion to *F. tularensis* [21]. Three B.22 samples were found in other neighboring counties, one B.22 D4 type was found in Stockholm and two B.22 samples with D25 & D32 subtypes were found in counties W and S (Figure 1B). The B.22 samples located in multiple counties may reflect very recent dispersal events, which is supported by the MLVA subtype data, or errors in epidemiological records.

The B.22 SNP provides a striking line of evidence that supports the pattern that closely genetically related samples tend to have closer geographic proximity despite the complex phylogeographic landscape found in Sweden. Extrapolating from this model, SNPs that are highly specific to the sequenced genome may be found most frequently among samples recovered from the same location where the reference genome originated. Such SNPs would be both relationally and geographically informative and, therefore, useful in identifying a likely place of infection. That said, due to the rapid dispersal of *F. tularensis*, the geographical attribution of the B.22 SNP may be time limited.

The results of the present study indicate that SNP typing schemes, designed from geographically informative SNPs, combined with a MLVA typing scheme have the potential to be used as a standalone typing method in outbreak investigations. However, since our attempts to correlate geographic origin of the samples to specific genetic types did not generate a clear cut result despite employing a canSNP typing scheme that provided high genetic resolution we conclude that whole-genome sequencing (WGS) would be the most appropriate tool for effectively linking specific genetic type to geography. Comparing the WGS of an unknown sample to WGS databases of archived Swedish strains maximizes the likelihood of revealing those rare geographically informative SNPs among genetic near matches. This insight may prove useful for future epidemiological investigation practices.

## Materials and Methods

### Clinical samples

The Public Health Agency of Sweden receives continuously clinical specimens for primary diagnostics of *F. tularensis* from physicians. All samples were received during 1994 to 2010 from patients of both genders, varying ages (1–89 years), and from diverse regions in Sweden (Table S1). Since tularaemia is a noticeable disease in Sweden, cases had been reported to the Swedish National Surveillance System by the treating physician. If no likely place of exposure had been included, the patients were contacted. We extracted *F. tularensis* subspecies *holarctica* DNA from patients suffering from ulceroglandular tularaemia: 3 complex clinical samples taken directly from the wound site and 124 cultured samples. Genomic DNA was prepared using two commercially available DNA extraction kits, the QIAamp tissue protocol (Qiagen, Stockholm, Sweden) for samples from 1994 to 2005 and the NucliSens magnetic extraction protocol (Biomérieux, Gothenburg, Sweden) for *F. tularensis* samples and wound specimens from 2006–2010.

### canSNP

CanSNP analysis using 26 previously published assays was performed on the 127 clinical samples as described (Figure 1A) [6], [11], [12].

### MLVA

The markers Ft-M3, Ft-M6, Ft-M20, Ft-M22 and Ft-M24 were amplified as described [7]. The forward primers (Invitrogen Life Technologies, Paisley, United Kingdom), Ft-M3 and Ft-M20 were fluorescent labelled with NED, Ft-M6 and Ft-M24 with 6-FAM, and HEX for Ft-M22 respectively. PCR was performed in 12,5 µl reaction mixture containing 10 mM Tris-HCl, pH 8.3, 50 mM KCl, 2,5 mM MgCl_2_, 0.5 U AmpliTaq DNA polymerase (Applied Biosystems, Stockholm, Sweden), 0,1 mM deoxynucleoside triphosphates, 0,6 µM of each primer with the addition of 0,5–1,0 ng template. The reaction mixture was incubated at 94°C for 5 minutes and then cycled at 94°C for 30 s, 58°C for 30 s, and 72°C for 30 s, and finally at 72°C for 5 minutes. Amplicons were diluted 30 times and pooled into two combinations a) Ft-M3, Ft-M6, and Ft-M22 and b) Ft-M20, and Ft-M24, respectively. 1 µl of each pool was analysed with capillary electrophoresis (3130 Genetic Analyzer, POP7-polymer, and. GeneScanTM-500 ROX TM size standards, Applied Biosystems, Stockholm, Sweden). The GeneMapper (Applied Biosystems) software was used to determine the size of the amplicons and to calculate the number of repeats at each VNTR marker. The diversity (D) for each VNTR marker was calculated as D = 1-∑(allele frequency)^2^ [17].

### PFGE

PFGE analysis was performed on *F. tularensis* samples [18]. Agarose plugs were sliced and incubated in 10 U of restriction enzyme *PmeI* (Biolabs, New England) for 3 hours at 37°C. Electrophoresis was performed in 1% agarose with a switch time of 1.79 to 10.71 s at 6 V/cm for 24 hours at 14°C. *Salmonella enterica* serotype Braenderup strain H9812 restricted with *Xba*I was used for gel normalization. Gels were stained with gel red and gel images captured by using a Gel Doc 1000 imager (Bio-Rad).

PFGE images were analysed using Bionumerics v 6.01 (Applied Maths, Sint-Martens-Latem, Belgium). Unique PFGE patterns were analysed and compared manually for band polymorphism.

### Geomapping

To obtain phylogeographic patterns we mapped the phylogenetic groups on a geographic map of Sweden at the county level resolution (Figure 1B). Of the 127 patients, 24 were excluded from this analysis due to unknown or uncertain location of exposure or lack of genotype information. The study protocol was approved by the Regional Ethical Review Board in Stockholm (# 2008/1020-31/2).

## Supporting Information

Table S1 ***F. tularensis*** **clinical specimens from Sweden included in this study.**
(XLSX)
